# Seeing eye-to-eye: Social gaze interactions influence gaze direction identification

**DOI:** 10.3758/s13414-019-01671-1

**Published:** 2019-07-15

**Authors:** S. Gareth Edwards, Andrew P. Bayliss

**Affiliations:** School of Psychology, University of East Anglia, Lawrence Stenhouse Building, Norwich, NR4 7TJ UK

**Keywords:** Gaze perception, Joint attention, Eye contact

## Abstract

We tested whether gaze direction identification of individual faces can be modulated by prior social gaze encounters. In two experiments, participants first completed a joint-gaze learning task using a saccade/antisaccade paradigm. Participants would encounter some ‘joint-gaze faces’ that would consistently look at the participants saccade goal before participants looked there (Experiment 1) or would follow the participants gaze to the target (Experiment 2). ‘Non-joint-gaze faces’ would consistently look in the opposite direction. Participants then completed a second task in which they judged the gaze direction of the faces they had previously encountered. Participants were less likely to erroneously report faces with slightly deviated gaze as looking directly at them if the face had previously never engaged in joint gaze with them. However, this bias was only present when those faces had *looked first* (Experiment 1) and not when the faces *looked after* participants (Experiment 2). Comparing these data with gaze identification responses of a control group that did not complete any joint-gaze learning phase revealed that the difference in gaze identification in Experiment 1 is likely driven by a lowering of direct gaze bias in response to non-joint-gaze faces. Thus, previous joint-gaze experiences can affect gaze direction judgements at an identity-specific level. However, this modulation may rely on the socio-cognitive information available from viewing other’s initiation behaviours, especially when they fail to engage in social contact.

Social interaction is fundamental to the human experience, and, consequently, humans are experts at using the social information conveyed by the eyes of others in order to learn and interact (see Emery, 2000; Frischen, Bayliss, & Tipper, 2007, for reviews). People are particularly sensitive to being looked at (*direct gaze*), which signals an upcoming interaction (Hietanen, Leppänen, Peltola, Linna-aho, & Ruuhiala, 2008). Interestingly, when we expect to be interacted with, we overreport being looked at (Stoyanova, Ewbank, & Calder, 2010). Perhaps the most common gaze-based interaction that we engage in is *joint attention*, whereby we look towards the same referent as another person (Moore & Dunham, 1995). We can learn from prior joint-gaze encounters and deploy our social attention resources according to the previous behaviour of given individuals (Dalmaso, Edwards, & Bayliss, 2016). Thus, we can form expectations about the value of particular people’s social attention cues based on their prior behaviour, as we have formed expectancies about how they will behave. The present work investigated whether expectations about the behaviour of specific individuals, developed from prior interaction experience, would similarly lead to a bias to report being looked at by those faces, which signals that those faces are attempting to initiate an interaction with us.

Humans are highly skilled at judging where other people are looking, using geometric cues to accurately determine the locus of regard of a conspecific (Doherty, McIntyre & Langton, 2015; Jenkins, Beaver, & Calder, 2006). Seminal work by Perrett and colleagues with monkeys, and later Calder and colleagues’ work with human participants, have greatly advanced our understanding of gaze coding and implicated a specialised neuroarchitecture subserving this human proficiency (see Nummenmaa & Calder, 2009, for review). Calder et al. (2007; see also Carlin, Calder, Kriegeskorte, Nili, & Rowe, 2011) showed human anterior superior temporal sulcus (aSTS) cells selectively code for leftward and rightward gaze, a finding in accord with those from earlier single-cell recordings in macaque brains (Perrett, Hietanen, Oram, Benson, & Rolls, 1992). Behavioural work also supports the notion of direction-specific gaze coding. An elegant demonstration of this is drawn from adaptation studies showing that repeated exposure to gaze deviated to the left will result in compromised judgements of leftward gaze (but not rightward), as the system habituates to the repeated leftward gaze signal (e.g. Calder, Jenkins, Cassel, & Clifford, 2008; Jenkins et al., 2006).

One of the most salient forms of another’s gaze is *direct gaze*, where we are being looked at. *Direct gaze* captures attention and signals approach (Hietanen et al., 2008; Hamilton, 2016; Senju & Hasegawa, 2005). The importance of direct gaze as a social attention cue was elegantly exemplified by the demonstration that the human gaze perception system is biased towards interpreting gaze as being direct; under conditions of perceptual uncertainty, we will assume we are being looked at (Mareschal, Calder, & Clifford, 2013a; see also Mareschal, Calder, Dadds, & Clifford, 2013b). Such a bias is advantageous, as it helps us avoid the potentially costly mistake of missing a direct gaze signal.

The range of nondirect gaze that is perceived as direct has been termed the ‘cone of gaze’ (Gamer & Hecht, 2007). Interestingly, this bias for direct gaze has been shown to be modulated by individual differences relating to the judger (e.g. social anxiety: Gamer, Hecht, Seipp, & Hiller, 2011), physical characteristics of the face (e.g. attractiveness: Kloth, Altmann, & Schweinberger, 2011) and by other cues of self-relevance (Stoyanova et al., 2010). Particularly pertinent to the current work, Stoyanova et al. (2010) found that participants more readily reported being looked at when they simultaneously heard their own name being called. This could suggest that our perceptual bias of assuming direct gaze is heightened when we expect to be interacted with. However, rather than implying that the social attention system can inform the gaze perception system rapidly and dynamically, this could instead reflect a response bias to report direct gaze when a highly co-occurrent cue (hearing one’s own name) occurs, rather than a perceptual bias per se.

Gaze that is not directed at us can be particularly informative, as we can use it to assess what the gazer is looking at and thinking about, and even engage with the gazer by looking at the same referent (Joint attention; Moore & Dunham, 1995; see Frischen et al., 2007, for review). Joint attention involves two interactive partners that each take a distinct role as either the initiator (gaze leader) or the responder (gaze follower) in the interaction, with the experience of each person being overlapping yet distinct (Mundy & Newell, 2007; see also Bayliss et al., 2013). Both following and leading gaze can have online consequences for attention (e.g. Edwards, Stephenson, Dalmaso, & Bayliss, 2015; see Frischen et al., 2007, for review). Many of the features that modulate direct gaze perception outlined above can also modulate the way in which we respond to the social cues of others in a joint-gaze encounter (e.g. physical facial characteristics: Jones et al., 2009; individual differences: Bayliss & Tipper, 2005). However, person knowledge can also alter the way in which we respond to the social attention cues of others (e.g. Carraro et al., 2017). It is to our knowledge untested whether person knowledge can similarly affect gaze perception responses to individual faces.

We can learn from joint attention encounters: Socio-evaluative judgements of joint-gaze partners and referent objects are sensitive to the quality of the joint-gaze encounter—we prefer objects and agents from joint over non-joint encounters (e.g. Bayliss, Paul, Cannon & Tipper, 2006; Bayliss & Tipper, 2006; Grynszpan, Martin, & Fossati, 2017). Learning from joint-gaze encounters can even alter our future behaviour towards joint-gaze partners such that we will entrust more money to reliable joint-gaze partners than to non-joint-gaze faces (Rogers et al., 2014).

Dalmaso et al. (2016) showed that the learning that occurs during social attention encounters, operationalised as successful or unsuccessful joint-gaze episodes, can affect how our social attention system responds to the social cues of those that we have previously interacted with. Participants completed a training phase where they had to saccade towards a peripheral cue if it appeared green, but (anti) saccaded away from the cue if it appeared red. Task-irrelevant faces appeared at the centre of the screen on each trial, with some faces paired to saccade trials and other faces paired to antisaccade trials. The faces would always display averted gaze towards the saccade/antisaccade peripheral cue, meaning that faces paired to saccade trials would always engage in joint gaze with a participant, while the faces paired to antisaccade trials would never engage in joint gaze with a participant. In a second gaze-cueing task, the same faces from the training phase appeared at the centre of the screen and looked left or right. These gaze cues would validly or invalidly (with equal probability) cue participants to the location of a to-be-discriminated target.

Illustrating that person knowledge can permeate the social attention system and inform the way in which our own social attention is deployed based on our knowledge of how a given identity has behaved previously, Dalmaso et al. (2016) found that in the second gaze-cueing task, participants responded differently to the gaze cues of faces depending on whether that face had or had not engaged in joint gaze with the participant during the training phase. Interestingly, across multiple experiments Dalmaso et al. also manipulated the joint-gaze role of the participant—whether they were the gaze leader or gaze follower. Not only were participants sensitive to the quality of previous encounters (whether joint gaze had occurred with a particular identity), but the social attention responses of a participant on reencountering a joint or non-joint-gaze face also varied based on the role in which that face was encountered (follower or leader). Specifically, when participants had been the gaze follower, they would later respond ‘normally’—with rapid gaze cueing at a 200 ms stimulus-onset asynchrony (SOA), but no gaze cueing later at a 1,200-ms SOA—to the gaze cues of joint-gaze faces but showed a delayed orienting response to non-joint-gaze faces (gaze cueing at the later but not earlier SOA). In contrast, when participants had been the gaze leader, they responded more than ‘normal’ to the social gaze cues of non-joint-gaze faces with gaze cueing emerging at both SOAs but did not show a gaze cueing of attention response at either SOA to joint-gaze faces. While the mechanisms behind each of these findings are far from established, taken together this evidence does show that our expectations about the behaviour of a given individual can guide how we interpret and evaluate their social cues (see Capozzi & Ristic, 2018).

Given that joint-gaze encounters appear to alter expectations regarding the interaction behaviour of prior joint-gaze partners, one might predict that the perception of other cues that signal interaction, such as direct gaze, might also be sensitive to the identity-specific social learning occurring in joint-gaze encounters. That is, we could predict that the bias to report direct gaze from a given identity might vary in accordance to the perceived likelihood of interaction from that particular individual. While connecting the disparate prior literature may make this suggestion seem reasonable, such a finding would be theoretically striking: Firstly, an identity-specific modulation of gaze perception based on prior interactions would not only show that it is the higher levels of evaluation and interpretation that are affected by social learning from gaze-based interactions but also implicate that this socio-cognitive information can permeate gaze *perception* (see Capozzi & Ristic, 2018). Secondly, while a unidirectional link between the social attention and gaze-perception systems has been evidenced previously, our work may reveal whether this ‘direct link’ is bidirectional (Bayliss, Bartlett, Naughtin, & Kritikos, 2011).

We describe two experiments that assessed how prior joint-gaze encounters with specific individuals affect later gaze perception of those same identities. Participants first completed a joint-gaze learning task as either the gaze follower (Experiment 1) or gaze leader (Experiment 2), and then performed a second gaze-perception task where they made speeded gaze-direction identifications of the faces that they had previously encountered. We anticipated that in both experiments’ participants would be more expectant of interaction from those face identities that had previously engaged in joint gaze with them, and so show a larger bias to report direct gaze for these faces.

## Experiment 1: Gaze cueing and eye-gaze-direction identification

In order to assess whether the gaze-perception system is permeable to socio-cognitive information gleaned from previous joint-gaze interactions, we first turn to *gaze following*, which compared with *gaze leading* is the more thoroughly researched side of joint attention (see Frischen et al., 2007). After completing a learning phase in which certain face identities always, or never, gaze-cued participants to their saccade target, participants made speeded identification of the gaze direction of the faces they had previously encountered.

Participants were asked to identify the gaze direction of faces whose gaze could be direct, deviated to the left or right by 5°, or deviated to the left or right by 10°. Previous research implementing a similar gaze-discrimination task found that gaze deviated by 5° will be reported as direct on approximately 20%–40% of trials (Jenkins et al., 2006). We predicted that the direct gaze bias would be greater in response to faces that had previously engaged in joint gaze with participants than those faces that never engaged in joint gaze, reflecting a greater expectation of reengagement from these previously interactive faces.

### Method

In each experiment we report how we determined our sample size, all data exclusions (if any), all manipulations, and all measures collected (see Simmons, Nelson, & Simonsohn, 2012; see also LeBel et al., 2013).

#### Participants

We aimed for a sample of *n* = 20 to be comparable with other similar studies (e.g. Calder et al., 2008; Dalmaso et al., 2016), and did not perform an a priori power analysis to determine an appropriate sample size. We planned to use the effect size estimates from Experiment 1 to inform our target sample size for subsequent experiments. Twenty volunteers (mean age = 21.0 years, *SD* = 6.7, four were men) took part for course credit or payment. All reported corrected or corrected-to-normal vision. Questionnaires were administered for purposes of secondary exploratory analyses. The Autism-Spectrum Quotient (AQ; Baron-Cohen, Wheelwright, Skinner, Martin, & Clubley, 2001) was completed by all participants, and the final 15 recruited participants also completed the Social Phobia Inventory (SPIN; Connor et al., 2000).

#### Stimuli, materials, and apparatus

Four neutral greyscale photographs (two male and two female young adults; 9.6 cm × 12.8 cm) were taken from the set developed by Bayliss et al. (2011). Counterbalanced across participants, two of the identities (one of each gender) were designated as ‘joint-gaze faces’, appearing only on saccade trials, with the other two faces appearing only on antisaccade trials. The gaze-perception task presented the same faces with either direct gaze (0° aversion), averted 5° to the right or left, or 10° to the right or left. Truly direct gaze stimuli were presented on 20% of trials. A chin rest was used, and right eye position was tracked (Eyelink 1000, SR Research, Ontario, Canada; spatial resolution 0.1°, 500 Hz).

#### Design and procedure: Joint-gaze task (Dalmaso et al., 2016)

**Fig. 1:**
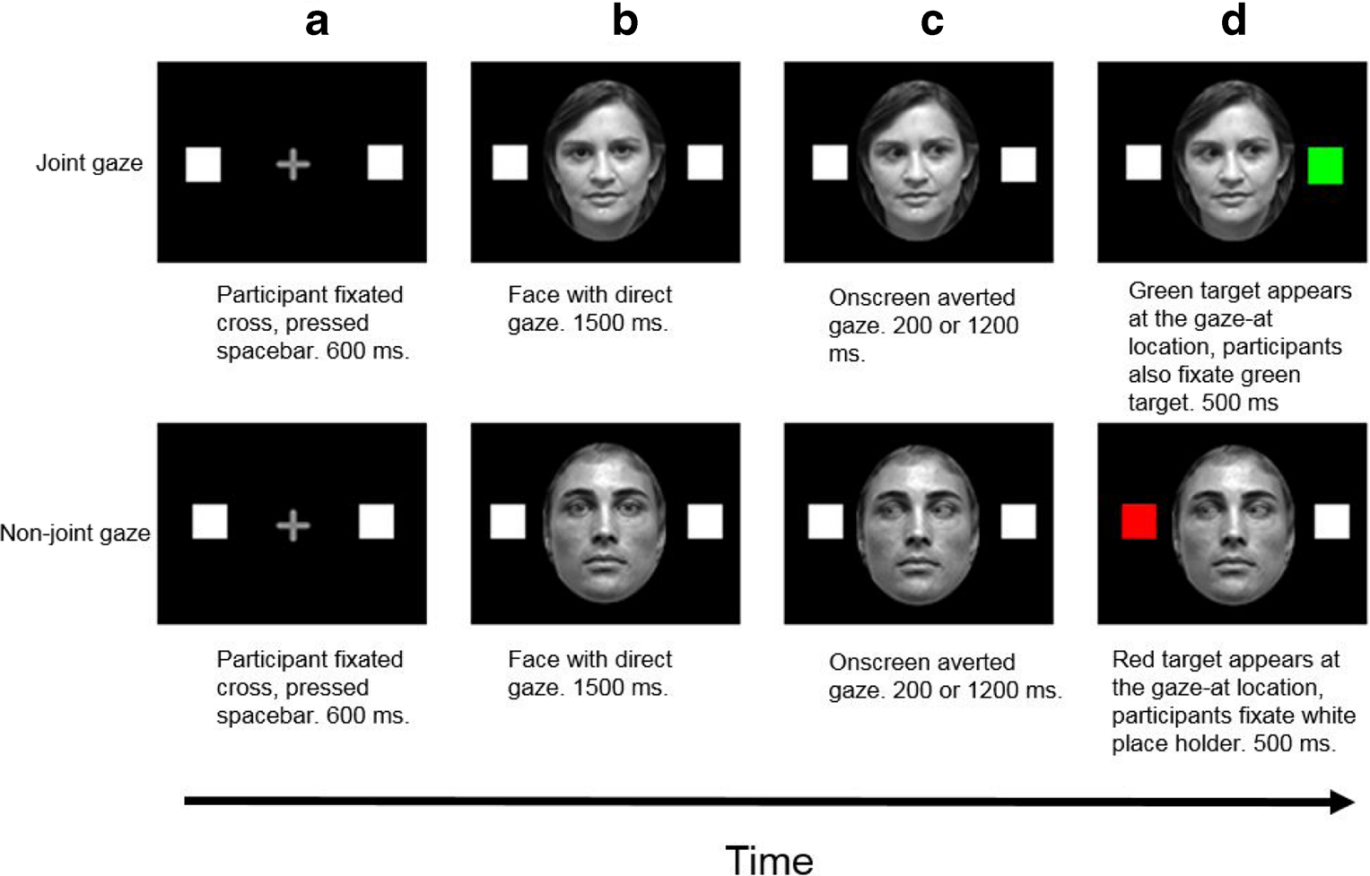
Example trials from Experiment 1, joint-gaze task. Participants first fixated the cross and pressed the spacebar (**a**), and 600 ms later, a face was displayed for 1,500 ms with direct gaze (**b**). Next, the same face displayed averted gaze to the left or right for either 200 or 1,200 ms (SOA; **c**). Finally, on joint-gaze trials, one of the placeholders turned green (upper panel), to which participants saccaded and fixated for 500 ms (**d**). For non-joint-gaze trials, the gazed-at placeholder turned red, indicating that participants should saccade to and fixate the opposite placeholder. Stimuli are not to scale. (Colour figure online)

Each trial began with a central grey fixation cross (0.8° height × 0.8° width) on a black background flanked by two white square placeholders (0.8° height × 0.8° width) placed 9.8° rightwards and leftwards from the cross. Participants fixated on the cross and pressed the space bar, which ensured correct fixation and preformed drift correction. After 600 milliseconds, the fixation cross was replaced by a central face with direct gaze (11° height × 8° width) for 1,500 ms. Next, the face would display averted gaze to the left or right for either 200 ms or 1,200 ms (SOA; see Dalmaso et al., 2016). Then, the placeholder that was ‘looked’ at by the on-screen face would change colour (red on antisaccade trials, green on saccade trials). Participants then made speeded saccades towards the green placeholder (saccade trials) or away from the red placeholder (antisaccade trials). The trial ended after 500 ms fixation of the correct placeholder (see Fig. 1). There were two blocks of 80 trials, with each trial type being presented an equal number of times, in a random order, per block. Participants were instructed to move their eyes as quickly and as accurately as possible and to ignore the faces and gaze direction.

#### Design and procedure: Gaze-perception task

**Fig. 2:**
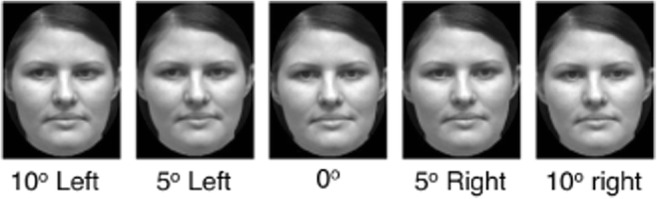
Example stimuli from the gaze-perception task. One face was displayed centrally, displaying one of five gaze directions; direct at participant (0° deviation), deviated by 5° to the left or right, or deviated by 10° to the left or right. Each face was displayed for 500 ms, during which time participants made speeded identification of gaze direction by pressing the 1, 2, or 3 key to indicate leftward, direct, or rightward gaze, respectively

A 2 (face type; joint gaze, non-joint gaze) × 2 (degree of averted gaze; 5°, 10°) design was implemented to assess the bias to incorrectly report direct gaze. Other trials presented gaze deviated by 0°, thus it was not always an error to report direct gaze. Each trial started with a central grey fixation cross, presented on a black background for 500 ms. Next, one of the faces from the joint-gaze task was shown for 500 ms with either direct gaze, averted by 5° or averted by 10° (see Fig. 2). Each of the four face identities were shown with each of the five gaze directions an equal number of times in a randomised order, across 280 trials. Participants were told to respond as quickly and accurately as they could by indicating the gaze direction of the face by pressing the 1, 2, or 3 keys to indicate leftward, direct, or rightward gaze, respectively, with their index, middle, and third finger of their preferred hand.

### Results

#### Joint-gaze task

The gaze-perception-task data, described below, is of primary interest. However, it is first important to inspect the joint-gaze-task data to ensure that the saccade/antisaccade task was successfully manipulating the participants eye-gaze fluency. Eye movement onset latency was defined as the time that elapsed from the instruction cue (colour change of the placeholder) to the initiation of the first saccade/antisaccade. The first saccade/antisaccade was defined as the first eye movement with a velocity exceeding 35°/s and an acceleration exceeding 9500°/s.^2^ Only saccades/antisaccades with a minimum amplitude of 1° were analysed (for a similar procedure, see Kuhn & Tipples, 2011). Trials containing blinks (5.5% of trials) were removed. Errors, namely trials in which the first saccade/antisaccade was in the opposite direction according to the instruction cue (11% of trials), were excluded from reaction time (RT) analysis and analysed separately. Outliers, defined as trials in which saccadic reaction times (sRT) were three standard deviations above or below participants mean (1.5% of trials), were discarded from analysis.

The percentages of errors for each participant in each condition were submitted to a 2 × 2 repeated-measures ANOVA with task (antisaccade vs. saccade) and SOA (200 ms vs. 1,200 ms) as within-subject factors. The main effect of task was significant, *F*(1, 19) = 12.82, *p* = .002, η_p_^2^ = .403, with more errors on antisaccade trials (*M* = 6.56%, *SD* = 7.08%) than on saccade trials (*M* = 3.09%, *SD* = 3.37%). The main effect of SOA was also significant, *F*(1, 19) = 15.71, *p* = .001, η_p_^2^ = .453, with more errors on trials with the shorter SOA (*M* = 6.13%, *SD* = 6.63%) than those with the longer SOA (*M* = 3.53%, *SD* = 4.50%). The Task × SOA interaction was also significant, *F*(1, 19) = 16.21, *p* = .001, η_p_^2^ = .460, due to a bigger difference in errors between trial types at the shorter (5.63%) than longer (1.31%) SOA. The equivalent ANOVA on the sRT data revealed a significant effect of trial type, *F*(1, 19) = 12.33, *p* = .002, η_p_^2^ = .394, with faster eye movements on saccade (315 ms) than antisaccade (351 ms) trials. The effect of SOA was not significant, *F*(1, 19) = .759, *p* = .394, η_p_^2^ = .038. The Trial Type × SOA interaction was significant, *F*(1, 19) = 7.93, *p* = .011, η_p_^2^ = .294, due to sRT advantage of saccade trials being larger at the shorter (300 ms vs 348 ms), than longer, SOA (330 ms vs 354 ms).

#### Gaze-perception task

**Fig. 3:**
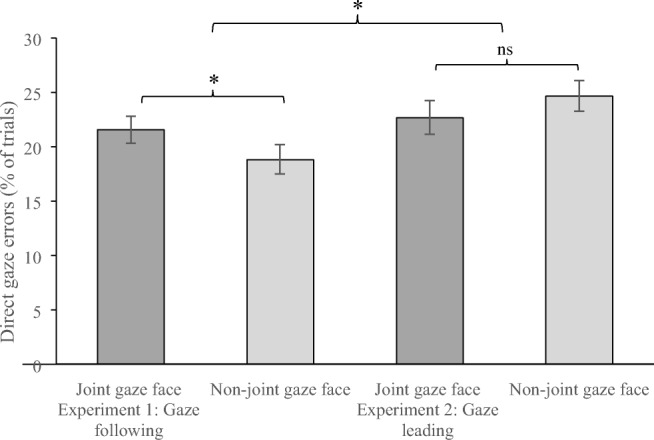
Percentage of trials in which participants incorrectly identified displayed on-screen gaze as ‘direct’ for faces that they had previously always—or never—engaged with in joint gaze, as either a gaze follower (Experiment 1) or gaze leader (Experiment 2). Error bars represent within-subject standard error of the mean (Loftus & Mason, 1994)

A 2 (trial type: saccade, antisaccade) × 2 (degree of averted gaze; 5°, 10°) ANOVA was conducted on the proportion of trials in which participants incorrectly indicated that the on-screen gaze was directed at them—pressing 2 to indicate ‘direct gaze’—when in fact the on-screen gaze was averted (but see Table 1 for all data including responses to gaze deviated by 0°). Here, reliably more direct-gaze errors were made in response to joint-gaze faces (21.56%) than to non-joint-gaze faces (18.80%), *F*(1, 19) = 5.46, *p* = .031, η_p_^2^ = .223 (see Fig. 3, Table 1). There were as expected, significantly more errors when the on-screen gaze was averted by 5° (34.58%) than by 10° (5.78%), *F*(1, 19) = 197.0, *p* < .001, η_p_^2^ = .912. The interaction was not significant, *F*(1, 19) = .761, *p* = .394, η_p_^2^ = .038.

**Table 1:** Percentage of trials judged as displaying direct gaze

	Joint-gaze faces			Non-joint-gaze faces		
Experiment 1—on-screen face cues participants						
Gaze deviation	0°	5°	10°	0°	5°	10°
Mean	84.17	36.46	6.67	84.38	32.71	4.90
*SD*	13.29	12.23	5.56	14.04	10.82	5.29
Experiment 2—on-screen face responds to participants						
Gaze deviation	0°	5°	10°	0°	5°	10°
Mean	83.33	36.69	8.55	83.33	38.65	8.46
*SD*	12.02	14.97	9.86	17.47	18.28	10.00

*Note. SD* denotes standard deviation

#### Questionnaires

Participants completed two questionnaires: the AQ and the SPIN. Here, neither self-reported autism-like traits, measured by the AQ, *r* = −.034, *n* = 20, *p* = .89, nor their self-reported social phobia level, measured by the SPIN, *r* = −.28, *n* = 15. *p* = .32, correlated with the differentiation of face type in the gaze-direction task (antisaccade face direct-gaze errors minus saccade face direct-gaze errors).

### Discussion

We asked participants to judge the gaze direction of faces that were either looking directly at them or slightly to the left or the right. We found that the tendency of participants to mistakenly attribute a photograph of a face as looking directly at them was influenced by the context in which the participant had previously encountered that face. Specifically, participants showed a smaller direct-gaze bias—making less erroneous ‘direct’ responses—on reencountering faces whose gaze they had previously not been able to follow, compared with faces whose gaze they had always followed.

It has previously been shown that the propensity to perceive averted gaze as direct can be affected by certain (perceptual) factors (e.g. Mareschal et al., 2013a). Extending on this prior work, we have shown that judgements about whether we are being looked at can be affected by socio-cognitive learning from prior joint-gaze encounters. Thus, this experiment presents, to our knowledge, the first illustration that the human gaze-perception system is sensitive to identity-specific information that is gleaned during gaze-based interactions, implicating both *memory* and *social attention* systems as interacting with the gaze-perception system.

Direct gaze is a communicative act (Senju & Johnson, 2009), an ostensive cue (Frith, 2008) that makes subsequent social attention cues more salient (e.g. Böckler, Knoblich, & Sebanz, 2011). Moreover, our perception of direct gaze is exaggerated by indicators of imminent interaction (e.g. Stoyanova et al., 2010). Therefore, the divergent direct-gaze bias for the two face types in the present study could be interpreted as indicating that expectations of interaction from these two face types have diverged, whereby participants are less expectant of signals of interaction from non-joint-gaze than from joint-gaze faces.

Participants might be less expectant of being interacted with by non-joint-gaze faces, compared with joint-gaze faces, as a result of being previously unable to end in a successful joint-gaze instance with those individuals. Indeed, it may be that exposure to the *spontaneous* gaze behaviour of others—as was the case here, with participants seeing on-screen gaze shifts before making their own—is necessary in order for such expectations of interaction to emerge: It is intuitive that predicting the likelihood of future initiation behaviours from others would be facilitated by exposure to these same types of behaviours from those social partners previously. One way to assess the above further would be to place participants as the gaze leader in the training phase. There would then be some faces that always or never engage in joint gaze with participants, but, crucially, the participant would not experience spontaneous initiation behaviour of their joint-gaze partners, but only experience how these identities respond to their own social cues. Thus, we may be able to tease apart whether the results of Experiment 1 relate to joint gaze per se or are specific to having experienced social initiation behaviours from specific individuals.

## Experiment 2: Gaze leading and eye-gaze direction identification

Experiment 1 had participants take the role of ‘follower’ in joint-attention episodes. However, where there is a follower, there is also a *gaze leader* (Bayliss et al., 2013). Gaze leading appears to be rewarding for humans (e.g. Schilbach et al., 2010), and as outlined in the introduction, leads to socio-evaluative outcomes that are comparable to those of following gaze. However, the information available to each of the leader and follower—from each other—in a joint-gaze encounter may differ in important ways.

In an archetypal joint-attention encounter, a gaze follower will have noticed that a conspecific has reoriented their social attention (e.g. eye gaze, pointing) and subsequently reorient their own attention towards the same referent object. The gaze follower can therefore infer whether the gaze leader intended for joint attention to ensue, and possibly learn to expect similar behaviours in the future from that individual (Dalmaso et al., 2016). However, the gaze leader has no direct experience of the gaze follower’s propensity to *initiate* interactions and can only learn about how likely the gaze follower is to *respond* to their own social cues (Dalmaso et al., 2016).

In order to assess whether gaze-leading encounters can affect gaze-perception judgements of prior joint-gaze partners, in Experiment 2 we flipped the procedure of the training phase such that the on-screen face would only display averted gaze once the participant had made their own imperative saccade. Thus, the participants would always have their gaze followed by some identities, but never by others. Crucially, participants would now only have experience of how the joint-gaze partners *responded* to them, but no exposure to their spontaneous gaze behaviours. Thus, if the quality of joint-gaze encounters per se modulates the expectation of interaction, Experiment 2 would replicate Experiment 1 with an attenuated direct-gaze bias for non-joint-gaze faces. However, if this perceptual modulation relies on having had access to the spontaneous initiation behaviours of the joint-gaze partners, Experiment 2 should result in little perceptual modulation.

### Method

#### Participants

A power analysis (G*Power; Faul, Erdfelder, Lang, & Buchner, 2007) using the effect size of η_p_^2^ = .223 from the main effect of face type from Experiment 1 found that *n* = 21, would deliver 1 − β power = 0.90 with an alpha of .05. We aimed for a sample on *n* = 21, stopping at *n* = 20 for convenience at the end of a run of booked testing sessions. Thus, in this experiment, a new sample of 20 volunteers (mean age = 19.0 years, *SD* = 1.4, two were males) participated for course credit or payment. All reported corrected or corrected-to-normal vision. The AQ was completed by all but one of the participants, and 18 completed the SPIN.

#### Stimuli and materials

Stimuli and materials were identical to those in Experiment 1.

#### Design and procedure: Joint-gaze task

**Fig. 4:**
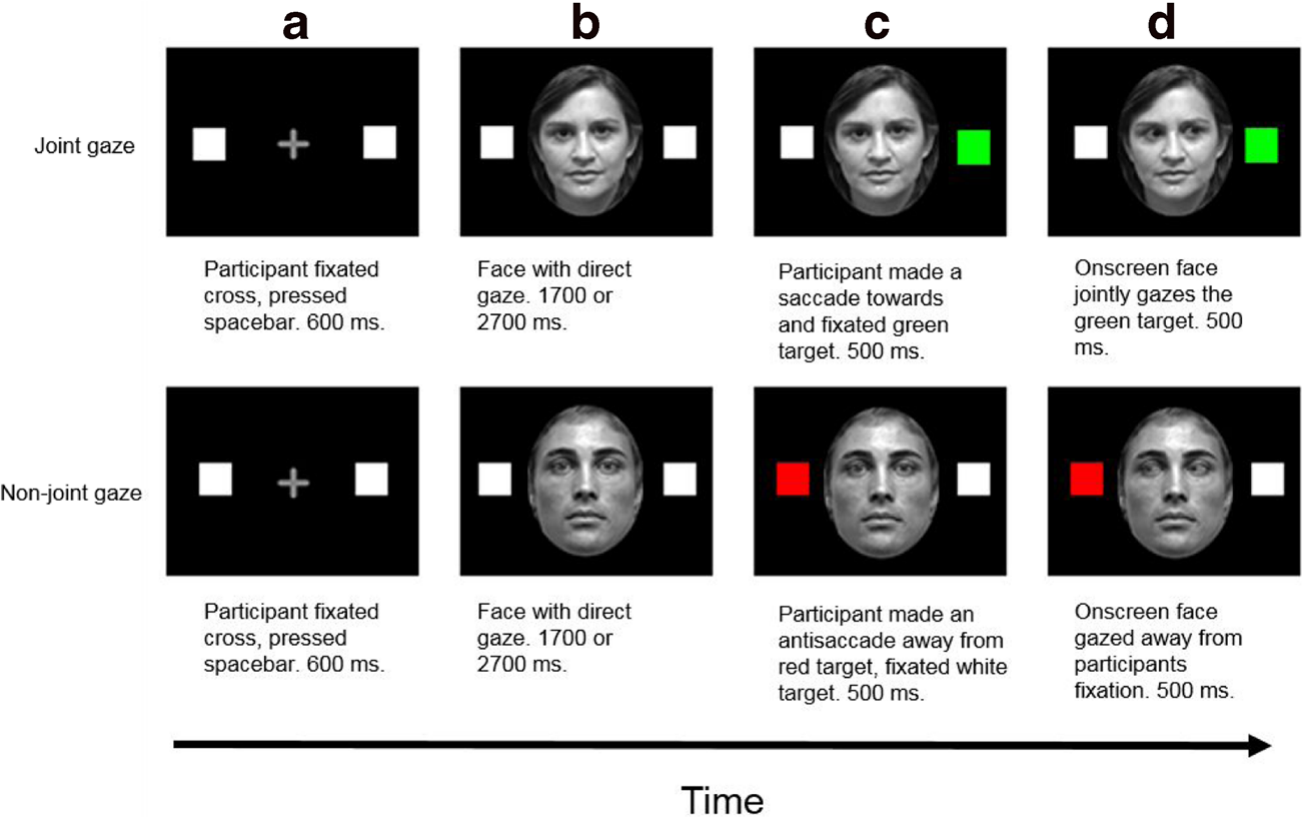
Example trials from Experiment 2, joint-gaze task. Participants first fixated the cross and pressed the spacebar (**a**); 600 ms later, a face was displayed for 1,700 ms or 2,700 ms (SOA) with direct gaze (**b**). On joint-gaze trials, one of the placeholders turned green (upper panel), to which participants saccaded and fixated for 500 ms, which triggered the on-screen face to display averted gaze also towards the green target (**d**). For non-joint-gaze trials, the sequence was identical (e.g. **a**, **b**), except that the target was red, to which participants made an antisaccade away from, fixating the opposite place holder for 500 ms (**c**), which triggered the on-screen gaze to ‘look’ away from the participants fixation and towards the red target. Stimuli are not to scale. (Colour figure online)

The design was identical to that of the joint-gaze task from Experiment 1, except that now the on-screen gaze would be displayed as averted *after* the participant’s own saccade (see Fig. 4). Therefore, the SOAs were adjusted so that the faces were shown for a comparable time to that of Experiment 1 (SOA: 1,700 ms; 2,700 ms).

#### Design and procedure: Gaze-perception task

The gaze-perception task was identical to that of Experiment 1.

### Results

Data analyses were performed as in Experiment 1.

#### Joint-gaze task

Trials containing blinks (2.18% of trials) were removed. Errors, namely trials in which the first saccade/antisaccade was in the opposite direction according to the instruction cue (12% of trials), were excluded from RT analysis and analysed separately. Outliers, defined as trials in which sRT were three standard deviations above or below participants mean (2.37% of trials), were discarded from analysis.

The percentages of errors for each participant in each condition were submitted to a 2 × 2 repeated-measures ANOVA with task (antisaccade vs saccade) and SOA (1,700 ms vs 2,700 ms) as within-subject factors. The main effect of task was significant, *F*(1, 19) = 10.83, *p* = .004, η_p_^2^ = .363, with more errors on antisaccade trials (*M* = 7.91%, *SD* = 7.19%) than on saccade trials (*M* = 3.50%, *SD* = 4.05%). Neither the main effect of SOA nor the Task × SOA interaction approached statistical significance (*F*s < 1.49, *p*s > .238).

The equivalent ANOVA on the sRT data revealed a significant effect of trial type, *F*(1, 19) = 35.90, *p* < .001, η_p_^2^ = .654, with faster eye movements on saccade (307 ms), than antisaccade (343 ms) trials. There was also a significant effect of SOA, *F*(1, 19) = 24.41, *p* < .001, η_p_^2^ = .562, due to faster eye movements when the SOA was longer (312 ms) than shorter (339 ms). The Trial Type × SOA interaction was also significant, *F*(1, 19) = 6.484, *p* = .020, η_p_^2^ = .254, due to sRT advantage of saccade trials being larger at the shorter (315 ms vs 362 ms) than at the longer (299 ms vs 324 ms) SOA.

#### Gaze-perception task

A 2 (trial type; saccade, antisaccade) × 2 (degree of averted gaze; 5°, 10°) ANOVA was conducted on the proportion of trials in which participants incorrectly indicated that the on-screen gaze was directed at them—pressing 2 to indicate ‘direct gaze’—when in fact the on-screen gaze was averted. There was no reliable difference in the percentage of direct-gaze errors made to each type of face (22.66% of trials for joint-gaze faces, 24.64% of trials for non-joint-gaze faces), *F*(1, 19) = 1.815, *p* = .194, η_p_^2^ = .087. There were, as expected, significantly more errors when the on-screen gaze was averted by 5° (37.71%) than by 10° (9.58%), *F*(1, 19) = 144.6, *p* < .001, η_p_^2^ = .884. There was no interaction, *F*(1, 19) = .075, *p* = .787, η_p_^2^ = .004.

#### Questionnaires

As in Experiment 1, neither AQ, *r* = −.30, *n* = 19, *p* = .22, nor SPIN, *r* = −.10, *n* = 18, *p* = .70, correlated with participants sensitivity to face type.

### Discussion

Participants made gaze-direction identification judgements of faces that they had previously encountered in a joint-gaze learning phase. In the learning phase, participants acted as the gaze leader; after participants had made their imperative eye movement towards or away from a peripheral cue, an on-screen face would ‘look’ to the same or opposite location to that which the participants were now fixating. Thus, some face identities would always follow the participants gaze and establish joint attention, while other face identities never established joint attention. Unlike Experiment 1, where participants had been the gaze follower in the learning phase interactions, in Experiment 2—where participants were gaze leaders—gaze direction perception did not differ between the two face types: Participants were equally likely to report being looked at by face identities that had always, or never, followed their gaze in the training phase. However, as interpreting null results can be difficult, below we analyse the two experiments together, with ‘experiment’ as a between-subjects variable, so that we can further assess the potential informative value of Experiment 2.

## Comparison between experiments

To directly compare the influence of following (Experiment 1) and leading (Experiment 2) joint-gaze bids on the bias to erroneously judge slightly deviated gaze as direct, we reanalysed the data, with ‘experiment’ as a between-subjects factor in a 2 (experiment) × 2 (face type) × 2 (degrees) ANOVA. The Face Type × Experiment interaction was significant, *F*(1, 38) = 6.322, *p* = .016, η_p_^2^ = .143, meaning that the performance of the participants in the two experiments was reliably different (see Fig. 2 and Table 1), with participants in Experiment 1 showing a significantly stronger impact of the first task on the second task than participants in Experiment 2. This suggests that one’s role in a joint-gaze encounter is critical to whether the gaze-perception system is affected, and that gaze-leading encounters do not modulate the bias to report direct gaze in the way that gaze-following encounters do. This suggests that rather than the joint-gaze end point being of primary importance, it may be that exposure to the initiation eye movements of others may be necessary in order to develop a bias regarding our perception of their gaze.

## General discussion

In two experiments we assessed the propensity to which participants would misperceive slightly deviated gaze as being direct gaze. The faces being judged had been encountered in a prior learning task. In Experiment 1 the participant was the gaze follower and experienced that some face identities would always cue their attention to a saccade target—meaning participants gaze followed and established joint gaze, while other faces would always look away from where the participant had to look. In a second task, participants showed a reliably larger bias to misperceive slightly deviated gaze as direct in response to faces that had always cued their attention, compared with the non-joint-gaze faces. However, in Experiment 2, where participants acted as the gaze leader in the learning phase—with some faces always or never following participants’ gaze to establish joint gaze—no statistically reliable direct-gaze modulation between face types emerged. Thus we, to the best of our knowledge, show for the first time that the gaze-direction perception system is permeable to socio-cognitive information gleaned from prior gaze-based interactions with specific individuals. Further, our data highlight the importance of the social learning afforded by one’s role in a social gaze encounter.

It is particularly interesting that our between-experiment analysis confirmed that gaze-following and gaze-leading encounters had differing impacts on later gaze-perception judgments, as this suggests that it is not whether the end point of joint gaze is achieved that influences later gaze perception. Rather, these data may indicate that the information portrayed by spontaneous initiation gaze behaviours of others—to which a gaze follower has access from a gaze leader—are necessary for the social learning required to inform expectations of interaction from those individuals in the future.

We note here the results from a follow-up experiment that we conducted in response to a reviewer who pointed out that the interpretation of the present data would be greatly facilitated by knowing whether the effect we observe in Experiment 1 is driven by an increase in direct-gaze bias for joint-gaze faces, or a reduction in direct-gaze bias for non-joint-gaze faces. We initially had hypothesised the former, but indeed, eyeballing Fig. 3 suggests the latter. In order to inform us in this regard, we had a separate group of 28 student volunteers (mean age = 20.2 years, *SD* = 4.8, two were males) complete only the gaze-direction identification task, without having any previous experience of the prior gaze behaviour of the faces they were judging. Here, participants incorrectly reported direct gaze on 26.58% trials. Welch’s *t* tests comparing the direct gaze bias for the four face types from Experiments 1 and 2 with this baseline reveals that only direct-gaze responses to non-joint-gaze faces from gaze following encounters (Experiment 1) significantly differed (*p* = .002, all other *p*'s *ns*). That is, the effect we observed in Experiment 1 appears driven by a lowering of the direct-gaze bias in response to face identities who had previously made initiation eye movements which negated the possibility of engaging in joint gaze with them. Thus, we suggest that our participants in Experiment 1 were less expectant of interaction from faces that had previously made spontaneous initiation eye movements that meant that participants could not engage in joint gaze with them, and thus these faces were misperceived as looking at participants less than all other face types.

The diminished direct-gaze bias for non-joint-gaze faces (Experiment 1) could be interpreted as reflecting more accurate processing of these potentially deceptive faces (Bayliss & Tipper, 2006; Carraro et al., 2017; Rogers et al., 2014). Yet exploratory analysis of the current data showed that speed of processing (RTs) of gaze direction for the most ambiguous gaze deviation (5°) did not differ between face types (*F*s < .523, *p*s > .478), suggesting the current findings do not reflect low-level efficiency differences. Interestingly, it has recently been shown that ostracised participants become less likely to report direct gaze (Syrjämäki, Lyyra, & Hietanen 2018). Future work may therefore look to assess whether the current findings can be interpreted as related to an identity-specific form of ostracism. Indeed, we anticipate this to be a socially specific effect. It will also be interesting for future work to assess whether the propensity to report direct gaze from specific identities can be increased, or whether it can only be reduced, as we have illustrated here.

Gaze-direction judgements of faces that had or had not previously followed the gaze of a participant (Experiment 2) did not reliably differ. However, numerically, the direct-gaze bias was larger in response to non-joint-gaze faces, and the effect size was nontrivial. As a reviewer suggested, this may indicate a smaller and opposite effect occurring in Experiment 2, relative to Experiment 1. Yet we feel that the data as presented currently are sufficient to draw the conclusion that (a) gaze-following encounters can influence subsequent gaze perception, and (b) that gaze-leading encounters do not do this in the same manner. Still, we note here, for completeness, that future work employing a greatly increased sample could evaluate the extent to which the data of Experiment 2 may reflect a weaker and opposite effect to that of Experiment 1.

Our data, showing that learning from social gaze interactions can modulate gaze perception, suggest that the previously reported ‘direct link’ between gaze perception and social attention systems is bidirectional (cf. Bayliss et al., 2011). Our data fit well with the ‘communicative intention detector’ and ‘fast-track modulation’ model of eye contact (see Senju & Johnson, 2009). Notably, our data clearly evidence the hypothesised links between gaze-direction coding in the anterior superior temporal sulcus, intentionality attribution by the posterior superior temporal sulcus and medial prefrontal cortex, and face identity coding in the fusiform gyrus (Frith & Frith, 2006; George, Driver, & Dolan, 2001; Johnson, 2005).

It is noteworthy that the imperative saccade task for a gaze leader necessitated participants fixating the referent object *prior* to the on-screen gaze aversion, meaning participants could only process the gaze-response peripherally (Experiment 2). Conversely, a participant who was following gaze could foveate the face as its gaze reoriented. The extent to which foveal processing of gaze reorientation is necessary for social gaze to affect gaze-direction judgements is therefore unclear. However, given that peripheral averted gaze detection would be necessary for real-world joint-gaze navigation—and experimentally has been shown to capture attention, influence subsequent eye movements and choice behaviour, and modulate subsequent social attention responses (e.g. Bayliss et al., 2013; Dalmaso et al., 2016; Edwards et al., 2015)—we suspect that the functional difference between gaze leading and gaze following is the key differentiating factor between experiments rather than any low-level perceptual difference relating to the ability to foveate the cue. Nevertheless, future work could directly assess this by manipulating whether participants are allowed, in the training phase, to ‘check back’ by fixating the on-screen face after the joint-gaze encounter is concluded.

Taken together, the current work presents what we believe to be the first evidence that the gaze-perception system is permeable to socio-cognitive information about individuals that we have previously interacted with. Thus, gaze perception and social attention systems interact bidirectionally, with memory and face-identity networks also connecting to the former. Moreover, our role as either the leader or follower in those previous interactions may be crucial to how we respond when reencountering individuals with whom we have previously interacted. Future investigations can aim to build a more thorough picture of this extended network including gaze processing, social attention, face-identity coding, and memory. These findings have implications for how we understand the relationship between gaze perception and joint attention, and on the role of interactions with specific individuals on these two elements of the social attention system.

### Author note

S. Gareth Edwards and Andrew P. Bayliss, School of Psychology, University of East Anglia.

S.G.E. and A.P.B. conceived the idea and designed the experiment. S.G.E. conducted the experiments, with assistance from Harriet Matthews and Peter Caudrey. S.G.E. analysed the data. Both authors interpreted the data and wrote the manuscript.

This research was supported by a University of East Anglia PhD Studentship to S.G.E. and a Leverhulme Trust Project Grant RPG-2016-173 to A.P.B.
